# Non-invasive mapping of ventricular action potential waveforms reconstructed from clinical unshielded magnetocardiography. Potential diagnostic application and current limitations^☆^

**DOI:** 10.1016/j.ahjo.2025.100561

**Published:** 2025-06-01

**Authors:** Riccardo Fenici, Marco Picerni, Peter Fenici, Donatella Brisinda

**Affiliations:** a Biomagnetism and Clinical Physiology International Center, Rome, Italy; b International School for Advanced Studies (SISSA), Trieste, Italy; c Catholic University of Sacred Heart, School of Medicine and Surgery, Rome, Italy; d Fondazione Policlinico Universitario Agostino Gemelli, IRCCS, Rome, Italy

**Keywords:** Magnetocardiography, Magnetic reconstruction of ventricular action potential, Monophasic action potential, Current arrow map, Non-invasive multimodal imaging of electrophysiologic events, Magnetoionography

## Abstract

**Objective:**

To evaluate the feasibility and limitations of reconstructing ventricular action potential waveforms using non-invasive, unshielded magnetocardiographic mapping (uMCG), highlighting differences between healthy individuals and patients, even at the current level of precision.

**Methods:**

Clinical uMCG was performed using a 36-channel DC-SQUID system. The mathematical reconstruction method developed by Kandori et al. was applied to derive reconstructed ventricular action potential waveforms (rVAPw) from uMCG data in 10 healthy volunteers and 12 patients with various cardiac abnormalities. In four cases, simultaneous recordings of uMCG and right ventricular monophasic action potentials (RVMAP) were obtained using an amagnetic catheter technique.

**Results:**

Reconstruction of rVAPw from uMCG signals was feasible in all subjects. Waveforms derived from 90-s averaged uMCG signals were comparable to those obtained with 300-s averages. The rVAPw closely matched the simultaneously recorded RVMAP waveforms. Compared to healthy individuals, patients showed a significant prolongation of rVAPw phase-0 (p < 0.01) and a trend toward increased total duration (p = 0.06), demonstrating the method's sensitivity to electrophysiological abnormalities.

**Conclusions:**

While incomplete rVAPw at some MCG mapping sites reflects the current spatial resolution limitations of the uMCG array, the close alignment between rVAPw and RVMAP recordings suggests that 90-s uMCG acquisitions may suffice for reliable, non-invasive imaging of ventricular action potentials in clinical practice. These findings support further development of MCG technology as a medical device uniquely suited to bridge experimental and clinical applications by enabling non-invasive rVAPw mapping in patients. Future improvements in sensor technology, mathematical modelling, and multimodal imaging may allow for near-cellular spatial resolution.

## 1 Introduction

Diagnosing the mechanisms underlying cardiac arrhythmias, evaluating the efficacy of antiarrhythmic drugs, and managing the cardiotoxic effects of non-cardiac pharmacological treatments remain major clinical challenges, particularly when chronic, life-saving therapy is required, as in oncology [1,2]. Since the 1960s, efforts to bridge the gap between experimental electrophysiology and clinical electrocardiography have led to key innovations, including catheter-based His bundle electrogram recordings [[3], [4], [5]], programmed cardiac pacing, and clinical recording of monophasic action potentials (MAPs) [6]. However, due to their invasive nature, these techniques are unsuitable for repeated monitoring.

In contrast, magnetocardiography (MCG), introduced around the same time [7], offers a noninvasive alternative with the potential for quick, repeatable electrophysiological assessments. It provides additional insights beyond traditional electrocardiography [8,9]. The utility of MCG has been progressively demonstrated through pioneering experimental research at institutions such as MIT and Vanderbilt University [[10], [11], [12], [13]]. Recent clinical studies further confirm MCG's diagnostic accuracy in detecting myocardial ischemia [[14], [15], [16], [17], [18], [19], [20], [21]] and assessing arrhythmogenic risk [22,23].

Despite these advances, a comprehensive mechanistic explanation for how a few seconds of resting-state MCG mapping can yield predictive accuracy comparable to interventional ECG testing is still lacking [9]. A plausible explanation lies in the direct relationship between cardiac magnetic fields and underlying electrical currents. This was clinically demonstrated by Kandori et al., who successfully reconstructed the ventricular action potential waveform (rVAPw) from noninvasive MCG mapping in a patient with a Type-I long QT (LQT) ECG pattern [24].

These preliminary findings demonstrated that the rVAPw derived from MCG data closely mirrored the right ventricular monophasic action potential (RVMAP), which had been invasively recorded at a site anatomically adjacent to the region of MCG signal abnormality. Importantly, the presence of an abnormal current arrow map (CAM) distribution at this location provided compelling evidence of MCG's potential to deliver novel, non-invasive insights into cardiac electrophysiology.

Indeed, Kandori's method is grounded in the relationship between current density and the magnetic field, as described by the Ampère-Maxwell law, and the relationship between current and voltage, as defined by Ohm's law. However, reconstructing the transmembrane action potential waveform from MCG data presents inherent challenges. This is because the transmembrane action potential is a localized event at the level of individual cardiomyocytes, whereas the MCG signal represents a spatially averaged current density generated by an indeterminate volume of underlying cardiac tissue. As such, any attempt to reconstruct transmembrane action potentials from MCG data may yield only a rough and indirect approximation.

To explore strategies for addressing these limitations, we leveraged a unique subset of our clinical MCG database that includes simultaneous uMCG mapping and right ventricular (RV) monophasic action potential (MAP) recordings, obtained using the amagnetic catheter technique [25].

We hypothesized that this unique dataset could provide a more robust foundation for evaluating the clinical utility of the method proposed by Kandori et al., which had previously been tested in only a single reported patient. In that case, the MCG data were acquired in a magnetically shielded room, and the RVMAP was recorded during a separate invasive electrophysiological study session.

This pilot study aims to generate previously unavailable data to assess the feasibility and limitations of Kandori's method for potential clinical application. Specifically, we investigated:

the feasibility of reconstructing ventricular action potential waveforms from multichannel MCG data recorded in an unshielded hospital laboratory fully equipped for clinical interventional electrophysiology; the direct comparison between the magnetically reconstructed ventricular action potential waveform and the simultaneously recorded RVMAP in patients undergoing invasive electrophysiological studies for diagnostic purposes [26]; and the reproducibility of rVAPw reconstruction in a representative human sample, including healthy volunteers as well as patients with cardiomyopathies, intraventricular conduction abnormalities, or arrhythmias.

## 2 Methods

### 2.1 Study population

uMCG data from 10 healthy volunteers (mean age: 38.1 ± 9.8 years) and 12 patients (mean age: 52.8 ± 20.0 years) were retrospectively selected as exemplary cases of cardiomyopathy, intraventricular conduction abnormalities, including right bundle branch block (RBBB), left bundle branch block (LBBB), right ventricular preexcitation, Brugada type 1 ECG pattern, and various arrhythmias. All subjects underwent uMCG mapping between 2002 and 2006, and were selected based on the following criteria: (1) availability of at least two sequential uMCG recordings of different durations (90 and 300 s); (2) an optimal signal-to-noise ratio in the unshielded MCG recordings; and (3) documented informed consent for the retrospective use of clinical and uMCG data for research purposes. Patients with atrial flutter or fibrillation were not excluded; however, for this study, only uMCG data recorded during sinus rhythm were analyzed for magnetic action potential reconstruction.

Additionally, data from four patients who underwent simultaneous contactless uMCG mapping and right ventricular monophasic action potential recording during diagnostic electrophysiological studies were preliminarily analyzed to evaluate the alignment between magnetically reconstructed action potentials and invasively recorded MAP waveforms. All four patients were in sinus rhythm during both MCG mapping and RVMAP recording. Since uMCG was performed as an adjunct during clinically indicated electrophysiological studies, the availability of MCG recordings during pacing was dependent on the specific diagnostic protocol applied in each case, and not standardized across patients.

### 2.2 Equipment and measurement techniques

(more details in the Supplementary file)

#### 2.2.1 Unshielded MCG recording and Postprocessing

**Fig. 1:**
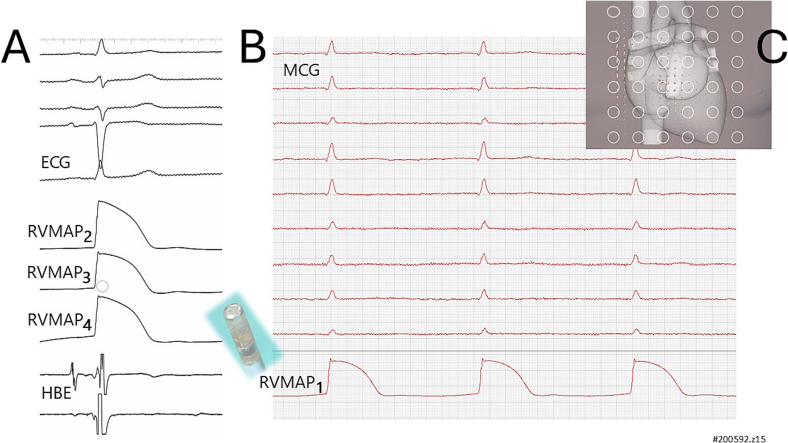
(A) Example of Ventricular MultiMAP recording with a single amagnetic catheter. (B) One MAP signal (MAP1) is connected to the CardioMag system to synchronize the MCG with the intracardiac electrophysiological mapping. (C) The three-dimensional model of the patient's heart is shown, with superimposed the recording sensor array and the current arrows map trajectory calculated after the QRS onset.

uMCG mapping was conducted on an outpatient basis using a 36-channel system (CardioMag Imaging, Inc., Schenectady, NY, USA) at the Catholic University's unshielded Biomagnetism Center (Suppl. Fig. 1a), which is fully equipped for interventional electrophysiology. The z-component of the cardiac magnetic field (Bz) was recorded using 36 direct current superconducting quantum interference device (DC-SQUID) sensors, each paired with a second-order axial gradiometer (baseline: 50 mm; pick-up coil diameter: 20 mm) [27] (Fig. 1).

All signals were digitally acquired with subjects in the supine position, from a 20 × 20 cm area over the anterior chest wall (Suppl. Fig. 1e). Following our laboratory's clinical research protocol, at least two sequential MCG recordings, one lasting 90 s and one 300 s, were routinely obtained. This protocol aimed to evaluate the repeatability of uMCG measurements and to assess the influence of the number of averaged cardiac cycles on the quality and reproducibility of uMCG analyses [28].

Post-processing of the uMCG signals included adaptive digital filtering to eliminate 50 Hz power line interference, followed by time averaging over either 90 or 300 s to enhance the signal-to-noise ratio before reconstruction of the time-varying magnetic field dynamics.

The CAM, which illustrates cardiac current flow patterns without requiring the solution of a nonlinear inverse problem, was computed according to the method described by Hosaka and Cohen [29] and visualized into a 3D heart model tailored to the individual patient's size by fitting in the model the patients' heart and chest measurements acquired with markers corresponding to the uMCG sensors' position (Fig. 1C).

**Fig. 2:**
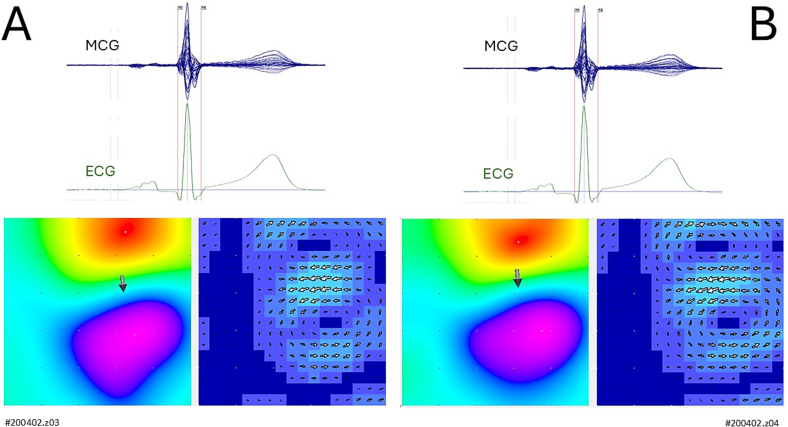
Examples of magnetic field distribution, effective magnetic dipole inverse localization, and current arrow map, computed at the onset of the QRS complex, showing the reproducibility between 90-s (A) and 300-s (B) MCG signal averages.

In addition, the magnetocardiographic inverse solution was computed using the effective magnetic dipole (EMD) model, to further analyze cardiac electrical activity with a temporal resolution of 1 millisecond (Fig. 2).

#### 2.2.2 The method for reconstruction of the ventricular action potential from uMCG signals

To reconstruct the ventricular action potential waveform from averaged uMCG data, the method described by Kandori et al. [24] was applied. This method is based on the relationship between the magnetic field and the ionic currents (inward and outward) of the cell membrane, which occur during the depolarization and repolarization phases, respectively, and generate the waveforms of both the action potential and the ECG. The electrical propagation of the cell membrane was calculated using the heart conduction system model by Beeler and Reuter [30].

A detailed description of the analytic method is provided in the Supplement file. Briefly, the computation of the components “I_x_ ” and “I_y_” of the CAM, which is the cornerstone of Kandori's work,  is a particular case of the Ampère–Maxwell law, which describes the relationship between electric current density and the surrounding magnetic field.

The reconstruction of the current densities requires an approximation of the spatial derivatives of the magnetic field. To perform such approximations, a finite difference scheme was employed.

It is crucial to note that this approximation improves as the measurement grid becomes finer (in turn, the estimate becomes rougher if the sensors are too far away from one another).

Improvements in resolution would allow for more localized and accurate reconstructions of current flow patterns, which are expected to correlate more closely with localized electrophysiological events (in this case, the RVMAP).

#### 2.2.3 Ventricular MultiMAP recording with the amagnetic catheter

The multipurpose amagnetic catheter for MCG-compatible simultaneous multiple MAP (MultiMAP) recording [25,31,32] features a variable number of non-polarizable amagnetic electrodes (Fig. 1A and B) and can simultaneously record four monophasic action potentials and perform local cardiac pacing and was preliminarily tested both in phantoms and in patients [33,34] to develop the Magnetic Source Imaging (MSI) method [35]. High-resolution MultiMAP recordings were differentially amplified (bandwidth DC-500 Hz), digitized at 1 kHz (CardioLab GE Medical System) and stored on disk for further analysis.

#### 2.2.4 Custom software for automatic analysis of the rVAPw and RVMAP signals

The custom software to calculate rVAPw from MCG and to quantitatively analyze the RVMAP and rVAPw signals, automatically detects the baseline, upstroke, peak, and offset of the signals. It also calculates the RVMAP duration at 50 % (d50%) and 90 % (d90%) level of repolarization, as well as the duration of phase 0 for each MAP and RAP (at 50 % and 90 % level of repolarization), after normalization of the rVAPw and RVMAP amplitudes. In patients with simultaneous uMCG and MAP recordings, one of the four MAP analog signals was connected to the MCG mapping system to synchronize the CardioMag with the Cardiolab data acquisition and to trigger MCG signal averaging (Fig. 1B).

#### 2.2.5 Statistical analysis

Statistical analysis was carried out with SPSS (Version 21.0). Differences were assessed by Student's t-test (paired or unpaired, as appropriate). A p-value <0.05 was considered statistically significant.

The repeatability (i.e., by definition, the capability of the instrumentation to reproduce results from two subsequent uMCG mappings performed by the same operator, under the same conditions within a short period) of uMCG measurement in our clinical laboratory was previously assessed through the Coefficient of Variability (CV) between parameters for each subject [28].

## 3 Results

### 3.1 uMCG recording and ventricular action potential reconstruction

After adaptive COMB filtering of the 50 Hz power line noise, the signal-to-noise (S/N) ratio of the MCG signals recorded within the DC-250 Hz bandwidth was sufficiently high to allow beat-to-beat cardiac monitoring and magnetic field analysis in all patients selected for this study (Fig. 1B). The calculation of the magnetic field distribution, EMD localization, and the CAM showed high reproducibility between the 90-s and 300-s signal averages (Fig. 2A and B).

**Fig. 3:**
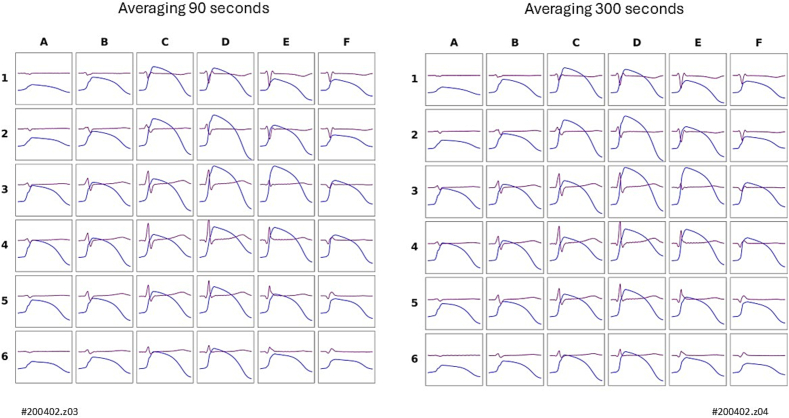
Example of reproducibility of rVAPw (blue) and superimposed averaged MCG waveforms (purple) obtained from 90-s (A) and 300-s (B) MCG signal averages (same healthy subject as ih Fig. 2). (For interpretation of the references to colour in this figure legend, the reader is referred to the web version of this article.)

The magnetic reconstruction of the action potential was also reproducible (Fig. 3) in all cases, with no significant differences in the rVAPw parameters, calculated at both the 90 % (avg. 9,8 ± 12,4 msec SD; p = 0.34) and 50 % (avg. 5,86 ± 5,96 msec SD; p = 0.67) repolarization levels and during phase 0 % (avg. 2,9 ± 2,6 msec SD; p = 0.7).

### 3.2 Monophasic action potential recordings

Four right ventricular monophasic action potentials were simultaneously recorded during uMCG mapping, with a single MultiMap amagnetic catheter in four patients, in sinus rhythm, without any catheter-induced magnetic artefact (Fig. 1B). When performing ventricular pacing with the same catheter, only two local MAPs were recorded.

### 3.3 Comparison between the magnetically reconstructed ventricular action potential and the RVMAP waveforms

Table 1 summarizes the comparison between the durations of the RVMAP and rVAPw, measured at 50 % (d50%) and 90 % (d90%) repolarization levels, as well as the corresponding phase 0 intervals, during sinus rhythm. The table presents data from two arbitrarily selected uMCG channels and from the channels exhibiting the maximum and minimum absolute differences between the MAP and rVAPw duration parameters. Due to technical limitations, RVMAP signals acquired during uMCG mapping in one of the four patients were degraded and thus excluded from analysis. As a result, Table 1 includes data from only three patients.

**Table 1:** Patients with simultaneous MCG mapping and right ventricular MultiMap recording.

Case #	Age	Diagnosis	Parameter	Channel		RVMAP (msec)	rVAPw (msec)	difference (msec)	% diff
200532.z13	75	RBBB, LAFB, 1° AVB, Syncope, DCM, Diabetes, Hypertension	d90	D4	Standard selection	356	362	6	1.66
				D5		356	365	9	2.47
				D1	Max diff	356	329	−27	−8.21
				A4	Min diff	356	358	2	0.56
			d50	D4	Standard selection	231	254	23	9.06
				D5		231	257	26	10.12
				C5	Max diff	231	329	98	29.79
				D3	Min diff	231	233	2	0.86
			ph 0 90	D4	Standard selection	105	122	17	13.93
				D5		105	111	6	5.41
				F6	Max diff	105	123	18	14.63
				C3	Min diff	105	105	0	0.00
			ph 0 50	D4	Standard selection	42	81	39	48.15
				D5		42	80	38	47.50
				F2	Max diff	42	88	46	52.27
				C6	Min diff	42	52	10	19.23
200592.z15	67	PAF, Syncope, LBBB (rate.dep), Hypertension	d90	D4	Standard selection	316	361	45	12.47
				D5		316	362	46	12.71
				E5	Max diff	316	363	47	12.95
				A3	Min diff	316	327	11	3.36
			d50	D4	Standard selection	242	293	51	17.41
				D5		242	256	14	5.47
				E1	Max diff	242	345	103	29.86
				A3	Min diff	242	240	−2	−0.83
			ph 0 90	D4	Standard selection	23	56	33	58.93
				D5		23	57	34	59.65
				A4	Max diff	23	58	35	60.34
				A1	Min diff	23	53	30	56.60
			ph 0 50	D4	Standard selection	13	38	25	65.79
				D5		13	37	24	64.86
				A4	Max diff	13	43	30	69.77
				A3	Min diff	13	35	22	62.86
200525.z8	72	RBBB, LAFB, 1° AVB, Lown4 Ventricular Arrhythmias	d90	D4	Standard selection	366	402	36	8.96
				D5		366	402	36	8.96
				C1	Max diff	366	406	40	9.85
				B1	Min diff	366	362	−4	−1.10
			d50	D4	Standard selection	273	274	1	0.36
				D5		273	280	7	2.50
				E3	Max diff	273	367	94	25.61
				D4	Min diff	273	274	1	0.36
			ph 0 90	D4	Standard selection	84	100	16	16.00
				D5		84	100	16	16.00
				A3	Max diff	84	104	20	19.23
				B6	Min diff	84	87	3	3.45
			ph 0 50	D4	Standard selection	65	60	−5	−8.33
				D5		65	58	−7	−12.07
				A6	Max diff	65	45	−20	−44.44
				D2	Min diff	65	65	0	0.00

RBBB: Right Bundle Branch Block; LBBB: Left Bundle Branch Block: LAFB: Left Anterior Fascicular Block; AVB: Atrio-ventricular Block; DCM: Dilated Cardiomyopathy; PAF Paroxysmal Atrial Fibrillation.

**Fig. 4:**
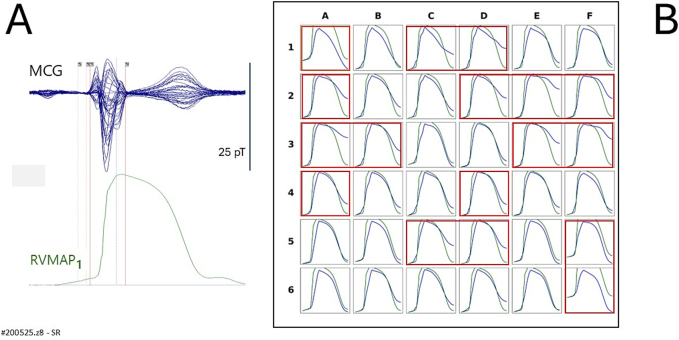
Patient # 200525.z8 in Table 1. (A) Simultaneous MCG (butterfly overlay) and RVMAP signals during the electrophysiologic study. (B) Comparison between RVMAP (green waveform) and rVAPw (blue waveform) The amplitude of the rVAPw is normalized to that of the RVMAP. A good agreement is observed at 19 recording positions, whereas some misalignment is evident at several MCG recording sites (highlighted by red squares). (For interpretation of the references to colour in this figure legend, the reader is referred to the web version of this article.)

Fig. 4A shows an overlay of the 36 unshielded MCG signals (butterfly plot) alongside the simultaneously recorded RVMAP obtained during the electrophysiological study (patient #200525.z8 of Table 1). Fig. 4B presents a spatial comparison in which the amplitude of the rVAPw, calculated at each MCG sensor location, is normalized to the amplitude of the corresponding RVMAP waveform. Direct comparison revealed satisfactory agreement between rVAPw and RVMAP waveforms at 19 of the mapping sites. However, in this patient, approximately 47 % of the rVAPw signals showed incomplete alignment with the RVMAP, particularly evident in the divergence of phase 3 repolarization timing.

**Fig. 5:**
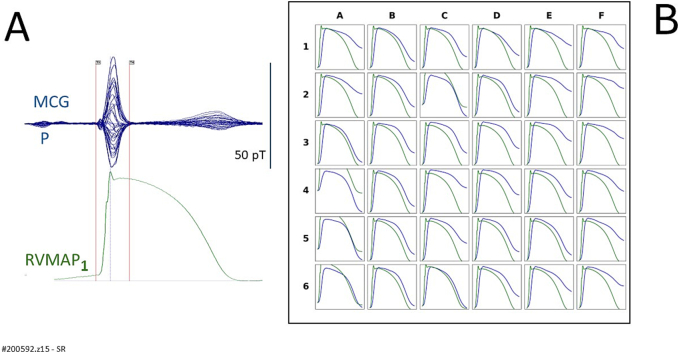
Patient #200592.z15, in Table 1. (A) Simultaneous MCG (butterfly overlay) and RVMAP recordings during the electrophysiological study in sinus rhythm. (B) Comparison between RVMAP (green waveform) and RAP (blue waveform). The amplitude of the rVAPw, normalized to that of the RVMAP, shows a good alignment at 11 recording positions. (For interpretation of the references to colour in this figure legend, the reader is referred to the web version of this article.)

**Fig. 6:**
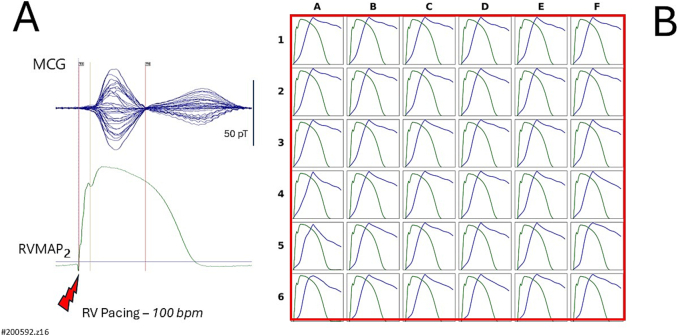
Example of unsuccessful action potential reconstruction during right ventricular pacing (same patient as in Fig. 5). (A) Simultaneous unshielded MCG signals (butterfly overlay) and right ventricular monophasic action potential (RVMAP) recordings. (B) In comparison to the RVMAP waveform, the reconstructed ventricular action potential waveform (rVAPw) appears unreliable, showing delayed and incomplete repolarization, specifically, the absence of a distinct phase 3 across all MCG recording sites.

**Fig. 7:**
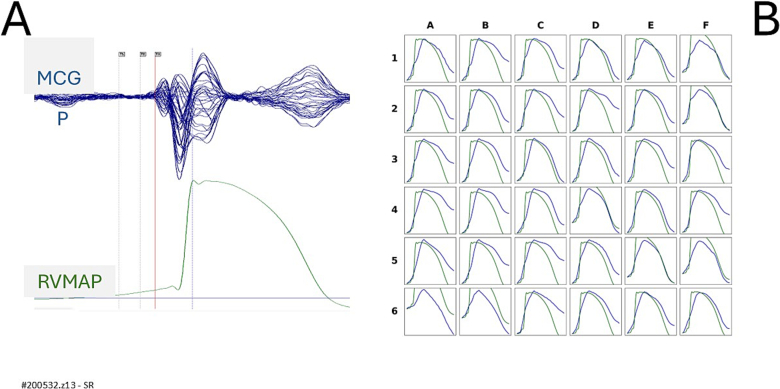
Patient #200532.z13 in Table 1. (A) Simultaneous unshielded MCG signals (butterfly overlay) and right ventricular monophasic action potential (RVMAP) recordings during sinus rhythm. (B) Comparison between the RVMAP (green waveform) and the reconstructed action potential (rVAPw; blue waveform), after amplitude normalization, shows a fair alignment, particularly in the overall waveform morphology and repolarization phase. (For interpretation of the references to colour in this figure legend, the reader is referred to the web version of this article.)

**Fig. 8:**
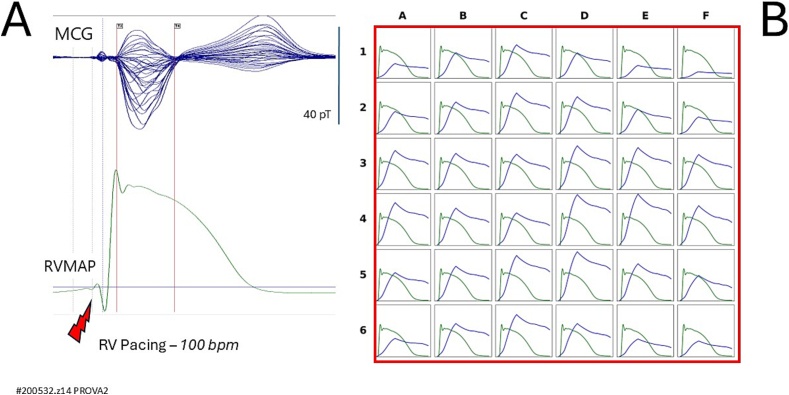
Same patient as in Fig. 7. (A) Simultaneous unshielded MCG signals (butterfly overlay) and right ventricular monophasic action potential (RVMAP) recordings during right ventricular pacing. (B) Reconstructed ventricular action potential waveforms (rVAPw) show unreliable results, with delayed and incomplete repolarization (specifically the absence of a clear phase 3 across all MCG recording sites).

In the two patients (#200592.z15 and #200532.z13) who underwent simultaneous recordings of uMCG and RVMAP during both sinus rhythm and right ventricular pacing, a good alignment between the reconstructed ventricular action potential waveform and the RVMAP was observed during sinus rhythm (Fig. 5, Fig. 7). However, during right ventricular pacing, the reconstruction of ventricular action potentials from uMCG signals proved unreliable (Fig. 6, Fig. 8). This was primarily due to delayed and incomplete ventricular repolarization, as evidenced by the absence of a clearly defined phase 3 at all MCG recording sites.

### 3.4 Magnetic action potential reconstruction in ambulatory patients and healthy volunteers

The data from reconstructed magnetic action potentials obtained from ambulatory unshielded MCG recordings in both patients with various cardiac abnormalities and healthy volunteers are summarized in Table 2, Table 3, respectively.

**Table 2:** Magnetic action potential reconstruction in ambulatory patients.

Case #	Age	Diagnosis	Channel	rVAPw duration (msec)		phase 0 duration (msec)	
				d90%	d50%	@90 %	@50 %
200402.Z1	72	RBBB, LAFB, 2°AVB, Hypertension	D4	357	234	114	61
			D5	359	272	115	66
200419.z6	49	DCM, LBBB	D4	403	296	114	83
			D5	436	322	116	88
200429.z4	71	LVH, Aortic valve insufficiency, PAF	D4	376	271	72	41
			D5	343	231	70	45
200501.z4	27	WPW (Right posterolater accessory pathway)	D4	463	370	78	45
			D5	465	282	80	45
200539.z2	30	Brugada Type 1 ECG pattern	D4	317	228	67	48
			D5	312	218	67	48
200422.z3	46	ARVD, Paroxysmal atrial flutter	D4	404	305	70	43
			D5	455	287	69	47
200487.z3	60	Ventricular arrhythmia (Lown 2), Hypertyroidism	D4	379	293	68	49
			D5	378	322	67	49
200634.z2	27	Syncope, Mitral Valve Prolapse, RBBB	D4	324	233	70	51
			D5	325	231	70	51
200492.z8	73	PAF, Syncope, LBBB (rate.dep), Hypertension	D4	358	214	108	72
			D5	309	204	106	67
200532.z2	75	RBBB, LAFB, 1° AVB, Syncope, DCM, Diabetes	D4	373	262	134	75
			D5	410	294	130	85
200525.z8	72	RBBB, LAFB, 1° AVB, Lown4 Ventricular Arrhythmias	D4	402	274	100	60
			D5	402	280	100	58
200482.z4	31	DCM	D4	352	285	74	57
			D5	347	268	73	59

RBBB: Right Bundle Branch Block; LBBB: Left Bundle Branch Block: LAFB: Left Anterior Fascicular Block; AVB: Atrio-ventricular Block; DCM: Dilated Cardiomyopathy; ARVD: Arrhythmogenic Right Ventricular Dysplasia, PAF: Paroxysmal Atrial Fibrillation.

**Table 3:** Magnetic action potential reconstruction in healthy volunteers.

Case #	Age	Diagnosis	Channel	rVAPw duration (msec)		phase 0 duration (msec)	
				d90%	d50%	@90 %	@50 %
200542.z8	51	fitness for sport activity	D4	336	251	61	40
			D5	336	247	63	45
200603.z2	42	Dizzines	D4	320	252	74	50
			D5	320	238	78	54
200545z1	54	fitness for sport activity	D4	372	299	87	51
			D5	369	276	83	51
200544.z2	35	fitness for sport activity	D4	421	321	70	50
			D5	428	315	69	49
200406.z3	34	healthy volunteer	D4	330	271	68	52
			D5	331	259	67	53
200405.z7	46	healthy volunteer	D4	428	339	70	52
			D5	429	330	72	51
200402.z3	27	healthy volunteer	D4	316	219	53	37
			D5	293	210	50	37
200616.z3	33	healthy volunteer	D4	286	197	53	29
			D5	320	213	52	31
200625.z1	25	healthy volunteer	D4	377	271	78	52
			D5	380	262	75	51
200613.z1	34	healthy volunteer	D4	327	254	70	49
			D5	328	251	67	51

rVAPw: reconstructed ventricular action potential waveform.

All recordings were performed during sinus rhythm. In both tables, individual values are reported for rVAPw duration at 90 % and 50 % repolarization levels (d90% and d50%), as well as for the duration of phase 0. These values were calculated from two arbitrarily selected MCG channels (D4 and D5) used for quantitative assessment.

In healthy subjects, the average (mean ± SD) durations of rVAPw repolarization and phase 0, measured at 50 % and 90 % of the waveform amplitude, were 352.4 ± 44.3 msec (d90%), 263.8 ± 39.3 msec (d50%), 68.0 ± 10.0 msec (phase 0 at d90%), and 46.8 ± 7.4 msec (phase 0 at d50%), respectively.

In patients, the corresponding mean values were 377.0 ± 4675 msec, 269.8 ± 40.1 msec, 88.8 ± 22.8 msec, and 58.8 ± 14.0 msec. Statistically significant differences were observed only in the phase 0 durations (p < 0.01), consistent with the conduction abnormalities in the patients' group.

**Fig. 9:**
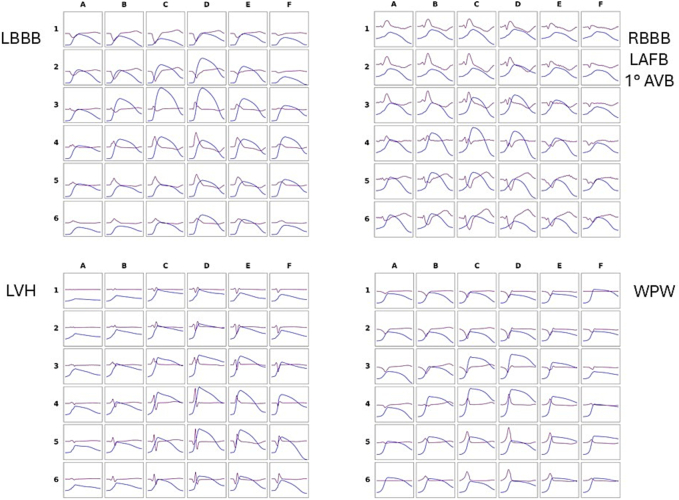
Examples of rVAPw reconstructed in patients with ventricular conduction abnormalities. (LBBB: left bundle branch block; RBBB: right bundle branch block, LAFB: Left anterior fascicular block; LVH: left ventricular hypertrophy; WPW: ventricular preexcitation (right posterolateral).

Fig. 3 illustrates an example of rVAPw reproducibility in a healthy subject. Fig. 9 presents examples of rVAPw from four patients with distinct types of ventricular depolarization abnormalities. Notably, the rVAPw in these cases shows progressively prolonged phase 0 duration, proportional to the degree of intraventricular conduction delay.

## 4 Discussion

In the 1960s, catheter-based recording of monophasic action potentials in humans was developed to bridge the gap between experimental cellular electrophysiology and the clinical diagnosis of the electrophysiological mechanisms underlying individual patients' arrhythmias [6]. With continuous advancements in catheter development and recording techniques, MAP recording became widely used to enhance the accuracy of clinical electrophysiological assessments, especially to study the ventricular repolarization reserve and restitution dynamics [4,25,[36], [37], [38], [39], [40]].

However, MAP recording requires invasive cardiac catheterization and is spatially limited by the mechanical constraints of catheter manipulation. In contrast, pioneering experimental studies have demonstrated that magnetocardiography can non-invasively detect cardiac electrophysiological phenomena, including subtle abnormalities that may be undetectable by standard electrocardiography [41] [8] [42,43]. As a contactless technique, MCG has long been envisioned as an ideal tool for non-invasive cardiac electrophysiology mapping in ambulatory settings, particularly for pre-interventional localization of arrhythmogenic foci. Beyond diagnosis, MCG has also shown potential for guiding interventional procedures, such as targeted endomyocardial biopsy or ablation of arrhythmogenic substrates that were prelocalized using MCG data [44].

A further refinement of this concept was introduced through a series of patents detailing the integration of MCG with single-catheter MultiMap recordings [25]. This hybrid approach supports minimally invasive, high-resolution electrophysiological studies performed with amagnetic catheters. The routine clinical implementation of MCG-guided MultiMap recordings in our laboratory has since been extensively documented [26,[45], [46], [47], [48], [49]].

These developments further support the growing momentum to explore the potential of uMCG mapping as a transformative tool for non-invasive, quasi-tissue-scale evaluation of cardiac electrophysiology in the clinical setting [9,36,50]. This vision is strongly reinforced by the groundbreaking study of Kandori et al. [24], who developed and patented a mathematical framework for the non-invasive reconstruction of ventricular action potential waveforms from cardiac magnetic field mapping.

Notably, that study [24] provided the first clinical demonstration that the reconstructed ventricular action potential waveform in a patient with a Long-QT type 1 (LQT1) ECG pattern exhibited abnormal features, including a potentially arrhythmogenic early afterdepolarization (EAD)-triggered activity. This finding was validated by comparison with a separately recorded right ventricular monophasic action potential waveform. However, upon closer examination of that report, particularly Fig. 9 in that reference [24], we observed that the reconstructed waveform appeared somewhat incomplete, with noticeable misalignment between the phase 0 take-off and phase 3 repolarization endpoints across several MCG mapping sites. This observation suggests methodological limitations that warrant further investigation, an area that, to the best of our knowledge, remains underexplored.

Accordingly, the present pilot study was designed to build on that original work by generating new insights made possible through the unique availability of simultaneous unshielded MCG mapping and MultiMAP recordings in our clinical database. The goal was to assess the current reliability and limitations of Kandori's reconstruction method and to provide a foundation for future hardware and software enhancements aimed at improving the clinical utility of this non-invasive approach.

From the direct comparison between rVAPw and simultaneously recorded RVMAP during sinus rhythm, we observed a generally good alignment between the two waveforms (Fig. 4B). However, at certain MCG recording sites, highlighted by red squares, the same type of waveform misalignment appreciable in Kandori et al.'s study [24] was also present, albeit to a lesser extent. The degree and prevalence of this misalignment varied across individuals, and due to the limited sample size, we were unable to definitively determine its cause. Possible contributing factors may include reduced magnetic signal strength at the peripheral regions of the sensor array, variations in local signal-to-noise ratio, or the limited spatial resolution and sensitivity of the 36-channel uMCG system used.

Similarly, we were unable to explain the failure of rVAPw reconstruction during right ventricular pacing (Figs. 6B and 8B), where the reconstructed waveforms lacked a clearly defined phase 3. This occurred despite the fact that, in the same patients, rVAPw and RVMAP waveforms showed fairly good alignment during sinus rhythm (Figs. 5B and 7B). These observations highlight specific limitations of the current system and underscore the need for further investigation into the technical and physiological factors affecting rVAPw reconstruction.

On the other hand, our results confirm the feasibility of rapid rVAPw reconstruction from 90-s averages of magnetic field mapping data in all subjects studied, demonstrating strong individual repeatability and reproducibility across magnetic field distribution patterns, current arrow maps, effective magnetic dipole localization dynamics, and rVAPw morphology (Fig. 2, Fig. 3). This is particularly encouraging given that all MCG recordings were acquired in a standard catheterisation laboratory using an unshielded system with only 36 channels, offering lower spatial resolution (4 cm vs. 2 cm sensor pitch) and reduced magnetic sensitivity compared to the system used by Kandori et al. in a magnetically shielded environment.

On average, rVAPw reconstruction from uMCG data was feasible at approximately 50 % of the MCG sensor positions, allowing for quantitative assessment of d90%, d50%, and phase 0 duration at these sites in all investigated subjects, highlighting differences between patients with advanced intraventricular conduction abnormalities and healthy individuals.

While these promising findings reinforce our view of MCG as a uniquely powerful tool for comprehensive, noninvasive clinical electrophysiological assessment [9], our data also highlight several limitations that must be addressed before the method proposed by Kandori et al. [24] can be translated into a reliable clinical tool for rapid and repeatable monitoring of cardiac electrophysiology and the potential cardiotoxic effects of chronic pharmacological therapies. Challenges related to spatial resolution, signal quality, and waveform completeness remain significant, underscoring the need for further advancements in sensor technology and signal processing techniques.

## 5 Limitations and lessons learned

Despite demonstrating the feasibility of reconstructing the ventricular action potential waveform with good individual reproducibility across repeated recordings, our findings also reveal significant limitations that must be addressed.

First, although direct comparison with simultaneously recorded RVMAP showed good alignment of the rVAPw at approximately 50 % of MCG recording sites, we observed occasional misalignments in phase 0 onset and/or phase 3 termination at randomly distributed sites across the sensor array. These discrepancies often manifested as a baseline mismatch between the rVAPw and RVMAP waveforms. The most frequent issue was the incomplete termination of the repolarization phase in the rVAPw or a misalignment of the baseline, which could not be resolved by manually extending the QTend parameter in the reconstruction software. This limitation mirrors a similar observation suggested by a detailed review of Kandori et al.'s Fig. 9 [24], and its underlying cause remains unclear.

Second, a comparable issue was consistently observed across all 36 recording sites in two patients during right ventricular pacing (Fig. 6, Fig. 8). In both cases, while rVAPw reconstruction during sinus rhythm had shown good alignment with RVMAP (Fig. 5, Fig. 7), reconstruction during pacing failed to produce complete repolarization waveforms, with a noticeable absence of phase 3 at every sensor location. The similarity in the rVAPw abnormalities observed during pacing in both patients suggests a shared underlying mechanism. One plausible hypothesis could be that ventricular pacing introduces electrotonic effects that alter local current density patterns in ways not accounted for by the reconstruction algorithm. Alternatively, the observed discrepancies may arise from limitations in the model assumptions—specifically, the use of uniform tissue conductivity and constant electrical resistance (set to 1), which may not hold true under the altered conduction dynamics induced by pacing. Dedicated experimental studies will be needed to test these possibilities and refine the computational model accordingly.

Indeed, incorporating variations in electrical resistance and conductivity into the modelling would be crucial, especially given the well-documented anisotropy of cardiac tissue under both physiological and pathological conditions [51,52]. While setting the resistance to a constant value of 1, as used in Kandori et al.'s model [24], may not significantly impact the overall conceptual outcome, more accurate computations would ideally account for the distinct conductivity properties of different types of cardiac myocytes or layers of cardiac tissue. However, implementing such refinements would require higher spatial resolution, which, as previously noted, remains a limitation of the current system. This challenge underscores the need for further advancements in both sensor technology and computational models to better reflect the complex physiological realities of cardiac electrophysiology.

Another key limitation is that the transmembrane action potential is a localized event that occurs at the level of individual cardiac myocytes, whereas the MCG signal reflects the spatially averaged current density generated by an undefined volume of underlying cardiac tissue. This fundamental difference in the source of the signals adds complexity to the accurate reconstruction of the ventricular action potential waveform from MCG data.

From a physiological standpoint, increasing spatial resolution would minimize the averaging effect of magnetic signals across multiple myocytes during measurement, highlighting the importance of independently detecting QRS intervals at each source site. Moreover, since the propagation of cardiac currents is inherently three-dimensional (3D), a higher density of sensors capable of simultaneously recording all three vector components of the cardiac magnetic field is crucial [53]. Achieving this increase in 3D spatial resolution would be the minimum requirement for accurate, high-resolution measurements of cardiac magnetic field dynamics and for distinguishing the underlying cardiac sources.

Hardware improvements must be complemented by advanced computational modelling that more accurately represents the heart's geometry and incorporates a deeper understanding of physiological electrical signals, such as ionic currents [[54], [55], [56]]. This would enable better simulations of how local action potentials from different regions of the heart contribute to the magnetic fields measured by MCG, thereby improving the calculation of the electric current (I) and enhancing the accuracy of action potential reconstruction. These advancements would ultimately provide more precise insights into local cardiac behaviour at each measurement site.

These partially negative findings may raise valid concerns regarding the limitations of reconstructing the action potential waveform from uMCG, as demonstrated in this study. However, they also underscore the need for further research to elucidate the mechanistic basis of the observed discrepancies. Such studies could provide valuable insights for refining MCG recording systems and computational models to address these limitations more effectively.

Despite these challenges, the preliminary demonstration that action potential reconstruction from uMCG is already feasible and repeatable at the clinical level represents a significant step forward. This lays the groundwork for the development of innovative MCG recording systems that can provide contactless magnetic mapping of cardiac action potentials, potentially on par with the results obtained in experimental animal models using optical mapping.

Moreover, the integration of recently reported MCG-derived Magnetoionography, which is capable of non-invasively measuring magnetic parameters sensitive to transmembrane calcium and potassium ion currents, as well as subcellular Ca^2+^ transients [57], strengthens our belief that, with advanced signal processing techniques, including deep learning algorithms [58], magnetocardiography has the potential to bridge the gap between experimental and clinical electrophysiology in a non-invasive manner.

## 6 Conclusions

Inspired by the seminal work of Kandori et al. [24], this study explored the feasibility and limitations of reconstructing ventricular action potential waveforms from non-invasive, unshielded magnetocardiographic mapping data, acquired in a clinical catheterization laboratory equipped for interventional electrophysiology, enabling simultaneous uMCG and monophasic action potential recordings.

The reproducibility of rVAPw obtained from 90-s averages of uMCG signals confirms that short-duration uMCG recordings may be sufficient for reliable action potential reconstruction in clinical settings.

Notably, in the three patients with simultaneous uMCG and RVMAP recordings, the magnetically reconstructed action potential waveforms align with simultaneous invasive RVMAP recordings, although a certain degree of partial misalignment can randomly occur at some of the MCG recording sites. Such partial misalignment at certain sites points to limitations in spatial resolution of the 36-channel uMCG system and/or the simplified method to calculate the MCG-derived current density used in this study.

Therefore, further development of novel dedicated hardware complemented by more advanced computational modelling, including more accurate simulations of ionic currents will be necessary to enhance the accuracy of electric current (“I”) calculations and, consequently, enable more localized action potential reconstruction and differentiation. Implementing this adjustment would substantially increase the computational complexity of the model, requiring more powerful computational resources. However, the increasing advancements in sensor density, mathematical modelling, and computational speed strengthen the case for near-future uMCG as a promising medical device for non-invasive clinical assessment of cardiac electrophysiology with quasi-cellular precision.

Finally, the multimodal integration of high-resolution MCG source localization with cardiac MRI or CT anatomical imaging [59,60] could significantly enhance the spatial localization of the reconstructed ventricular action potential waveform, providing an unrivalled non-invasive tool for clinical monitoring and evaluation of antiarrhythmic drugs efficacy, and to prevet and manage the cardiotoxic effects of chronic of life-saving pharmacological treatment in oncology patients [1,2,61], or multiple comorbidies [62].

To achieve this, efforts must focus on determining the optimal sensor density required to ensure the 3D spatial accuracy needed for high-resolution action potential reconstruction. Fortunately, experimental evidence already demonstrates that, with new sensor technologies, MCG can be recorded with millimeter-scale spatial accuracy [63]. Moreover, microfabrication advances have enabled the development of miniaturized quantum SERF magnetometers with an optimal magnetic sensitivity of 20 fT/Hz^1^/₂, which can be integrated at the chip scale for advanced magnetocardiography applications [64].

## CRediT authorship contribution statement

RF: Ideas; formulation or evolution of overarching research goals and aims; leadership responsibility for the research activity planning and execution; conducting the clinical research and experimental studies. Creation, writing of the manuscript draft; discussion, commentary, and revision in the pre-stages. Final revision of the manuscript.

MP Software development; implementation of mathematical algorithms for data analysis; data analysis. Critical revision of the manuscript draft and editing of the final version.

PF Critical revision of the manuscript draft and editing of the final version. Revision of the English form.

DB Responsible for the research activity planning and execution; conducting the research and investigation process, specifically performing the clinical recordings and patients' ambulatory control and follow-up. Data analysis and interpretation. Preparation and presentation of the material for publication; critical revision of the manuscript draft and editing of the final version

## Ethical statement

The authors ensure that they have written an entirely original work and that all reported work of others has been appropriately quoted and the relative reference cited as extensively as possible.

As a corresponding author, I ensure that all those who have made substantial contributions have been listed as co-authors and that all co-authors have seen and approved the final version of the paper and have agreed to its submission for publication.

Prof. Riccardo Fenici.

## Declaration of competing interest

The authors declare that they have no known competing financial interests or personal relationships that could have appeared to influence the work reported in this paper.

## Appendix A Supplementary data

Supplementary material Image 1
