# Supplementary material for: Non-invasive mapping of ventricular action potential waveforms reconstructed from clinical unshielded magnetocardiography. Potential diagnostic application and current limitations
Source: Am Heart J Plus. 2025 Jun 1;55:100561. doi: 10.1016/j.ahjo.2025.100561 (PMC12167455; doi:10.1016/j.ahjo.2025.100561)
Supplement: Supplementary file 1 — Supplementary material [file mmc1.docx]

# SUPPLEMENTARY FILE (Methods)

# Recording Equipments and Measurement Techniques

An overview of the clinical laboratory for integrated uushielded MCG mapping and interventional electrophysiology is shown in Suppl. Figure 1


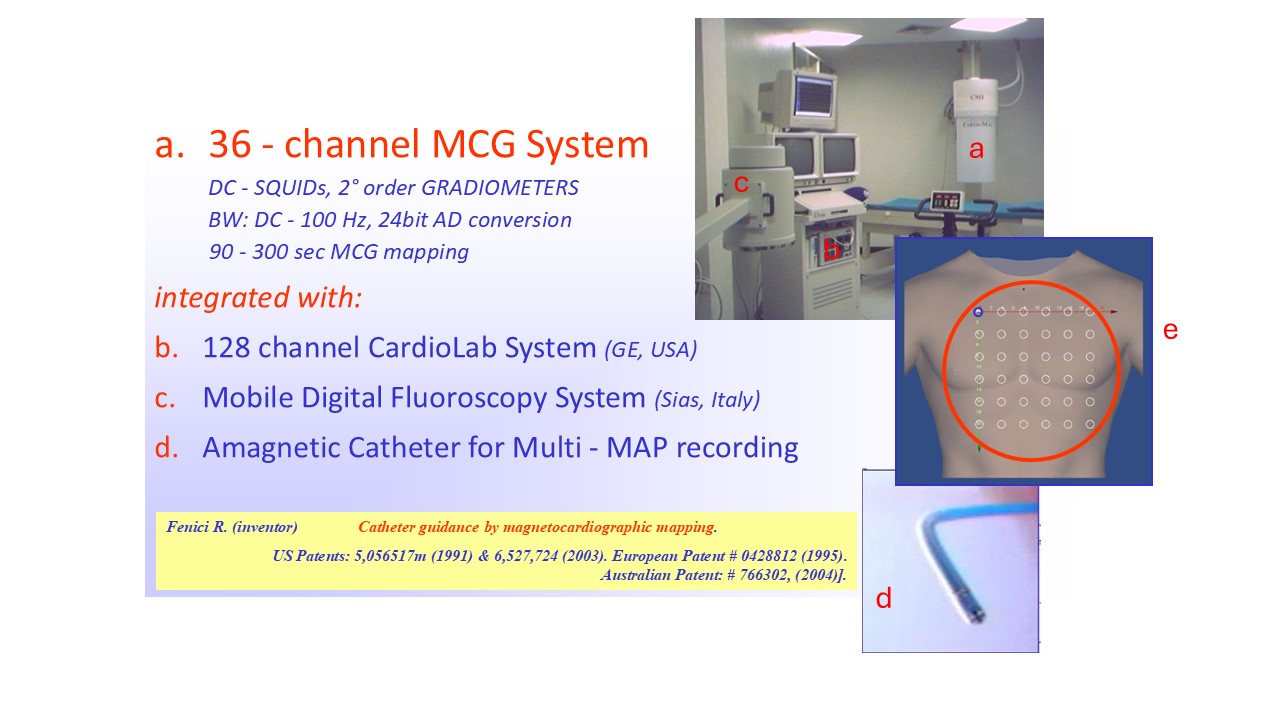


**Suppl. Figure 1.** Overview of the integrated equipments of the Biomagnetic Laboratory.

## Unshieled MCG Recording and postprocessing

uMCG mapping was performed on an outpatient basis using a 36-channel system (CardioMag Imaging, Inc., Schenectady, NY, USA) at the Catholic University’s unshielded Biomagnetism Center, which is fully equipped for interventional electrophysiology. The z-component of the cardiac magnetic field (Bz) was recorded with 36 direct current superconducting quantum interference device (DC-SQUID) sensors, each coupled to second-order axial gradiometers (with a 50-mm baseline and 20-mm pick-up coil diameter) (Figura 1a). These sensors were enclosed in a cylindrical cryostat and arranged in a square array with a 4 cm pitch (center-to-center). The system’s average effective sensitivity ranged from 30 to 50 fT/√Hz within the frequency range of clinical interest (DC–250 Hz), depending on the sensors' tuning accuracy and the level of environmental noise [1].

All signals were digitally recorded (24-bit conversion at a 1 kHz sampling frequency) in the supine position from a 20×20 cm surface of the anterior chest wall (Figure 1e). Two subsequent uMCG recordings, typically lasting 90 and 300 seconds, were performed on each subject to assess the effect of recording duration on the quality and repeatability of the uMCG signals [2].

Post-processing of the uMCG signals included digital filtering (using a selective COMB filter to remove 50 Hz power line noise), time averaging (over 90 or 300 seconds) to optimize the signal-to-noise ratio and reconstruction of time-variant magnetic field dynamics during sinus rhythm and cardiac pacing. The reference baseline was automatically defined before the P wave (in sinus rhythm) and before the QRS complex (during ventricular pacing).

The MCG inverse solution was calculated, with 1 msec time resolution, using the effective magnetic dipole (EMD) in a semi-infinite space model. The current arrow map, which provides a current pattern in the heart without having to solve a nonlinear inverse problem, was calculated according to Hosaka and Cohen [3] and was automatically visualized into a 3D heart model tailored to the individual patient’s size by fitting in the model the patients’ heart and chest measurements inferred from two orthogonal fluoroscopic, cardiac CT scan or MR images, acquired with markers corresponding to the uMCG sensors position (manuscript Figure1C).

## The method for reconstruction of the ventricular action potential from uMCG signals

To reconstruct the ventricular action potential waveform from averaged uMCG data, the method described by Kandori et al. [4] was applied. This method is based on the relationship between the ionic currents (inward and outward) of the cell membrane, which occur during the depolarization and repolarization phases, respectively, and generate the waveforms of both the action potential and the ECG.

The electrical propagation of the cell membrane was calculated using the heart conduction system model by Beeler and Reuter [5], along with the assumption that the MCG waveform reflects the spatial electrical activation of the heart.

The current density was computed by means of the Ampere-Maxwell formula. Since only the orthogonal component (B_z_) of the magnetic field was measured, we obtain the following formula for the components Ix and Iy of the CAM at each site:

$$I_{x}=\frac{\partial B_{z}}{\partial y}$$

and

$$I_{y}=-\frac{\partial B_{z}}{\partial x}.$$

Such partial derivatives were approximated by means of a finite difference scheme. At this stage, the importance of narrowing the approximation step, which means having a finer measurement grid, is evident, since a narrower grid would increase the precision of this approximation.

Improving this resolution would allow for more localized and accurate reconstructions of current flow patterns, which are expected to correlate more closely with localized electrophysiological events (in our case, the MAP).

The magnitude of the current vectors, representing the intensity of the current, was calculated^[[1]](#footnote-1)^ as their Euclidean norm:

$$\sqrt{{I_{x}}^{2}+{I_{y}}^{2}}$$

The CAM provides a dynamic, time-resolved visualization of the current pattern in the heart, bypassing the need to solve a nonlinear inverse problem.

Then, assuming a uniform cell-to-cell gap junction resistance (set to 1), the ventricular action potential waveform was reconstructed by summing and subtracting electrical currents during different phases of the cardiac cycle, as shown below.

During phase 0 (depolarization), currents from the onset to the end of the QRS complex (ventricular depolarization) are summed, starting from an initial value of zero and continuing until the end of the QRS (AP maximum). This summation reflects the rapid influx of Na+ ions that initiates and propagates the action potential.

During phases 1 to 3 (repolarization), currents are subtracted starting from the AP maximum value (at the end depolarization - QRS_end_) to the end of the T wave (T_end_). This subtraction represents the outward flow of K^+^ ions during ventricular repolarization, gradually returning the membrane to its resting potential.

Indeed, the stability of the results may partly stem from the method used to approximate electric current values. This approximation may be influenced by the large distance between measurement sites, particularly given the four-centimeter pitch of our uMCG systems. In particular, the approximated values may fail to fully capture the behaviour of magnetic field transients in regions where the magnetic field Bz exhibits sharp, localized peaks. This issue is especially noticeable when a magnetic field peak lies between two measurement sites. A higher spatial resolution would both prevent such occurences and the need for the aforementioned averaging procedure to compute the values of *I*. On the other hand, it is important to note that since the relationship between *“I”* and Bz is strictly localized, the calculation of the current sources located at the center of the sensors is not affected by the lower stability observed near boundary regions.

## Ventricular MultiMAP recording with the amagnetic catheter

The multipurpose amagnetic catheter for MCG-compatible simultaneous multiple right ventricular (RV) monophasic action potentials (MAP) (MultiMAP) recording is a patent of the Italian National Research Council [6]. The catheter is equipped with a variable number of non-polarizable amagnetic electrodes at the tip (Suppl. Figure 1d). In its simplified configuration, the catheter can simultaneously record four monophasic action potentials and perform local cardiac pacing.

The prototype of the amagnetic catheter was initially used to test and validate the accuracy of magnetocardiographic source localization, both experimentally in phantoms and clinically in patients [7], [8]. The 3D position of the catheter’s tip within the heart can be automatically detected and displayed using the Magnetic Source Imaging (MSI) method [9]. In modified versions, the catheter includes additional lumens for inserting steerable wires and optical fibers, making it suitable for laser ablation procedures.

High-resolution MultiMAP recordings, with an inter-MAP electrode distance of 1.2 mm, were differentially amplified (bandwidth DC-500 Hz), digitized at 1kHz (*CardioLab GE Medical System*) and stored on disk for further analysis. All tip electrodes were connected to the positive input of high-impedance DC-coupled optically isolated differential preamplifiers, while the reference electrode was connected to the negative input of the amplifier. With this recording setup, the MAP signals are oriented upward (manuscript Figure 1 A).

## Custom software for automatic analysis of the rVAPw and RVMAP signals

The custom software to calculate the rVAPw from MCG and to quantitatively analyze the RVMAP and rVAPw signals was developed in Python and automatically detects the baseline, the upstroke, peak and offset of the QRS signals. It also calculates the RVMAP duration at 50% (d50%) and 90% (d90%) level of repolarization, as well as the duration of phase 0 for each RVMAP and rVAPw (at 50% and 90% level of repolarization) after normalization of the rVAPw and RVMAP amplitudes. These measurements are independent of scaling factors and the selected baseline value, ensuring that they remain unaffected by the specific choice of the resistance factor (r = 1), as long as the uniformity assumption for the resistance value *“r”* holds.

In patients with simultaneous uMCG and RVMAP recordings, one of the four RVMAP analog signals was connected to the MCG mapping system to synchronize the *CardioMag* with the *Cardiolab* data acquisition and to trigger MCG signal averaging [10] (manuscript Figure 1B).

# REFERENCES

[1] Fenici R, Brisinda D, “First 36-channel System for Clinical Magnetocardiography in Unshielded Hospital Laboratory for Cardiac Electrophysiology.,” *Int. J. Bioelectromagn.*, vol. 5, no. 1, pp. 80–83, 2003.

[2] A. R. Sorbo, G. Lombardi, L. La Brocca, G. Guida, R. Fenici, and D. Brisinda, “Unshielded magnetocardiography: Repeatability and reproducibility of automatically estimated ventricular repolarization parameters in 204 healthy subjects,” *Ann. Noninvasive Electrocardiol.*, vol. 23, no. 3, pp. 1–12, 2018, doi: 10.1111/anec.12526.

[3] H. Hosaka and D. Cohen, “Part IV Visual determination of generators of the magnetocardiogram,” *J. Electrocardiol.*, vol. 9, no. 4, pp. 426–432, 1976, doi: https://doi.org/10.1016/S0022-0736(76)80043-X.

[4] A. Kandori *et al.*, “Reconstruction of action potential of repolarization in patients with congenital long-QT syndrome,” *Phys. Med. Biol.*, vol. 49, no. 10, pp. 2103–2115, 2004, doi: 10.1088/0031-9155/49/10/019.

[5] G. W. Beeler and H. Reuter, “Reconstruction of the action potential of ventricular myocardial fibres.,” *J. Physiol.*, vol. 268, no. 1, pp. 177–210, Jun. 1977, doi: 10.1113/jphysiol.1977.sp011853.

[6] Fenici Riccardo, “Catheter Guidance By Magnetocardiographic Mapping,” US 6,527,724 B1, 2003.

[7] R. Fenici *et al.*, “Magnetocardiographic pacemapping for nonfluoroscopic localization of intracardiac electrophysiology catheters.,” *Pacing Clin. Electrophysiol.*, vol. 21, no. 11 Pt 2, pp. 2492–2499, Nov. 1998.

[8] R. Fenici *et al.*, “Nonfluoroscopic localization of an amagnetic stimulation catheter by multichannel magnetocardiography.,” *Pacing Clin. Electrophysiol.*, vol. 22, no. 8, pp. 1210–1220, 1999.

[9] R. R. Fenici *et al.*, “High resolution MSI-guided multiple monophasic action potential mapping with a single amagnetic catheter,” in *Biomag 2000 : proceedings of 12th International Conference on Biomagnetism, August 13-17, 2000, Helsinki University of Technology, Espoo, Finland / edited by J. Nenonen, R. J. Ilmoniemi, T. Katila*, pp. 3–6.

[10] R. Fenici and D. Brisinda, “Bridging noninvasive and interventional electroanatomical imaging: role of magnetocardiography,” *J. Electrocardiol.*, vol. 40, no. 1 SUPPL., pp. 47–52, 2007, doi: 10.1016/j.jelectrocard.2006.10.032.

1. For sites distant from the boundary, these values are approximated by averaging over the values *I_x_* and *I_y_* obtained from multiple numerical methods [↑](#footnote-ref-1)
